# Transplantation sites for human and murine islets

**DOI:** 10.1007/s00125-017-4362-8

**Published:** 2017-07-22

**Authors:** Rebecca A. Stokes, Kim Cheng, Amit Lalwani, Michael M. Swarbrick, Helen E. Thomas, Thomas Loudovaris, Tom W. Kay, Wayne J. Hawthorne, Philip J. O’Connell, Jenny E. Gunton

**Affiliations:** 1 Centre for Diabetes, Obesity & Endocrinology, The Westmead Institute for Medical Research (WIMR), Room 2040, Level 2, Darcy Rd, Westmead Hospital, The University of Sydney, Sydney, NSW 2145 Australia; 2 Diabetes and Transcription Factors Group, Garvan Institute of Medical Research (GIMR), Sydney, NSW Australia; 3 National Pancreas Transplant Unit, University of Sydney, Westmead Hospital, Sydney, NSW Australia; 4 Faculty of Medicine, University of Sydney, Sydney, NSW Australia; 5 School of Medical Sciences, University of New South Wales, Australia, Kensington, NSW Australia; 6 St Vincent’s Institute, Melbourne, VIC Australia; 7 St Vincent’s Clinical School, University of New South Wales, Sydney, NSW Australia

**Keywords:** Experimental diabetes mellitus, Heterologous transplantation, Islets of Langerhans, Isogeneic transplantation

## Abstract

**Aims/hypothesis:**

Beta cell replacement is a potential cure for type 1 diabetes. In humans, islet transplants are currently infused into the liver via the portal vein, although this site has disadvantages. Here, we investigated alternative transplantation sites for human and murine islets in recipient mice, comparing the portal vein with quadriceps muscle and kidney, liver and spleen capsules.

**Methods:**

Murine islets were isolated from C57BL6/J mice and transplanted into syngeneic recipients. Human islets were isolated and transplanted into either severe combined immunodeficiency (SCID) or recombination-activating gene 1 (RAG-1) immunodeficient recipient mice. All recipient mice were 8–12 weeks of age and had been rendered diabetic (defined as blood glucose concentrations ≥20 mmol/l on two consecutive days before transplantation) by alloxan tetrahydrate treatment. Islets were transplanted into five different sites (portal vein, quadriceps muscle, kidney, liver and spleen capsules). Blood glucose concentrations were monitored twice weekly until mice were killed. Dose–response studies were also performed to determine the minimum number of islets required to cure diabetes (‘cure’ is defined for this study as random fed blood glucose of <15 mmol/l).

**Results:**

For transplantation of murine islets into the different sites, the kidney yielded 100% success, followed by muscle (70%), portal vein (60%), spleen capsule (29%) and liver capsule (0%). For human islets, transplantation into the kidney cured diabetes in 75–80% of recipient mice. Transplantation into muscle and portal vein had intermediate success (both 29% at 2000 islet equivalents), while transplantation into liver and spleen capsule failed (0%). With increased islet mass, success rates for muscle grafts improved to 52–56%.

**Conclusions/interpretation:**

For both human and murine islets, equivalent or superior glucose lowering results were obtained for transplantation into skeletal muscle, compared with the portal vein. Unfortunately, kidney grafts are not feasible in human recipients. Skeletal muscle offers easier access and greater potential for protocol biopsies. This study suggests that human trials of muscle as a transplant site may be warranted.

## Introduction

Pancreatic islet transplantation provides a potential cure for type 1 diabetes. Short-term and longer-term outcomes are improving [1–4] but most recipients still require islets from two or more donors to achieve insulin independence. Although the pancreas is conceptually attractive as the original ‘home’ of islets, it is not an easy or safe site [5].

Hepatic infusion via the portal vein is the currently accepted clinical site since the success of the Edmonton protocol. This is partly based on the theoretical advantages of the portal vein [6]. In a non-diabetic individual, circulating nutrient concentrations (including blood glucose levels) increase postprandially and the nutrients are delivered to the pancreas where beta cells respond by secreting insulin. Similarly, the liver is a conceptually attractive site for islet transplantation due to first-pass exposure to both nutrients and insulin [1, 7]. Early studies in mice reported success using the portal vein site [8] and subsequent refinements improved outcomes [3, 4, 9]. However, this site has disadvantages including the potential complications of portal hypertension, bleeding, portal vein thrombosis and hepatic ischaemia [10, 11]. These risks are low in the hands of experienced transplant centres with current protocols.

Another major problem with the portal vein is the rapid loss of many islets after transplantation. Contributing factors include instant blood-mediated inflammatory reaction, relative hypoxia and deleterious effects of immunosuppressive drugs which are absorbed from the gut (the liver is intermittently exposed to supra-therapeutic concentrations [2, 12–18]). Most grafts show gradual functional decline [9] and routine biopsies for graft monitoring are not feasible.

It is unclear to what degree the transplantation site influences outcomes. Few studies have compared the outcomes of human islet transplantation into different sites [19]. Mouse islets have been successfully transplanted into several sites [20] including the peritoneal cavity [21, 22], spleen [23, 24], portal vein [25, 26] and the kidney subcapsular space [27, 28]. However, there is a paucity of data concerning longer-term success rates [20]. Successful autotransplantation of islets into the muscle of pigs, dogs, rats, mice and, more recently, humans has been reported [19, 29–32].

We hypothesised that with its good arterial blood flow, and the successful autotransplantation cases mentioned above, skeletal muscle may be a viable site for human islet transplantation. In addition, if grafts were marked at placement, muscle would be amenable to low-risk protocol biopsies. Our study compares the outcomes for transplant sites including muscle, portal vein, and kidney-, liver- and spleen-capsule transplant sites in mouse and human islets. Most importantly, this study was performed under conditions that mimic human clinical protocols. In addition, increased islet dose experiments indicate improved outcomes for muscle grafts for both human and mouse transplants.

## Methods

### Ethics

Human islet studies were approved by the Western Sydney Local Health District Ethics Committee (AU RED LNR/15/WMEAD/386) and St Vincent’s Hospital Human Research Ethics Committee (H09/208). Animal studies were approved by the Western Sydney Local Health District Animal Ethics Committee and the Garvan Institute Animal Ethics Committee. Human pancreatic islets were purified using a modified Ricordi method [3, 33].

### Recipient animals

For syngeneic mouse islet transplants, donors and recipients (8–12 weeks old) were sex-matched and full MHC-matched (all C57BL/6) and transplanted at a one donor to one recipient ratio (220–250 islets per recipient), as previously reported [12]. Islet dose experiments used 1.5 donors or two donors per recipient. C57BL/6 mice were obtained from Australian Bio Resources (Moss Vale, NSW, Australia). Human islet recipients were immunodeficient mice (severe combined immunodeficiency [SCID] mouse model or mice deficient in recombination-activating gene 1 [RAG-1]), 8–12 weeks of age, obtained from the Animal Resources Centre (Canning Vale, WA, Australia) or Australian Bio Resources. We chose these recipients to avoid immunological rejection.

### Induction of diabetes in mice

Diabetes was induced in C57BL/6 recipients with intravenous alloxan tetrahydrate (Sigma-Aldrich, St Louis, MO, USA) at 110 mg/kg, and in SCID (Balb/C background) and RAG-1 (C57BL/6 background) mice with 75 mg/kg of alloxan. Alloxan was chosen due to a lower occurrence of beta cell regeneration. Diabetes was defined as blood glucose ≥20 mmol/l (≥360 mg/dl) for two or more consecutive days before transplantation and/or at the study completion. Mice without diabetes (blood glucose <20 mmol/l [<360 mg/dl]) at study end underwent graft removal to confirm diabetes recurrence (thus excluding endogenous beta cell regeneration). Graft removal was performed by nephrectomy, splenectomy, partial hepatectomy or myectomy, as appropriate.

### Islet isolation and transplantation

Islets from C57BL/6 mice were isolated and purified as previously reported [12, 13, 34]. They were counted, cultured overnight and transplanted within 24 h to mimic human islet transplantation [12]. Human islets were cultured overnight and transplanted into recipient mice using our previously reported adequate-mass model of 2000 islet equivalents (IEQ) per recipient [12].

We studied five transplant sites: kidney, spleen, liver, portal vein and muscle. Subsequent islet dose experiments tested kidney, muscle and portal vein, using either 3000 IEQ or 4000 IEQ per recipient. In each experiment, the kidney was the control and for every human donor at least one kidney transplant was performed. For each human donor, transplantation was performed at three or more sites (including the kidney control) to allow comparison between sites and to avoid the important confounder of inter-donor variability. Islets were kept suspended in media until immediately before transplantation to avoid compaction [35]. The islets were transplanted into anaesthetised mice (2% isoflurane maintenance plus oxygen) and were gently and linearly dispersed in each tissue location. In the portal vein, they were infused over 3–4 min.

Graft function was assessed by random fed blood glucose levels three or more times a week until either 28 days or long-term assessment 100 days after transplantation. Recipients that died during surgery were excluded. Mice with blood glucose >20 mmol/l were treated with daily insulin (0.5 U/kg) after glucose measurement. Depending on the transplant site, if a mouse was not frankly diabetic (blood glucose ≥20 mmol/l), nephrectomy, splenectomy, lobectomy or myectomy was performed and blood glucose levels were followed to confirm diabetes recurrence. If mice were clearly diabetic at study completion, they were not subjected to graft removal. Mice were arbitrarily considered ‘cured’ if random fed blood glucose levels were <15 mmol/l (see next section). In most cases, random fed levels were <8 mmol/l. Islet samples were randomised for both site and recipient. The experimenter was both islet isolator and surgeon and was therefore unable to be blind to group assignment; hence randomisation was carried out where possible. The same experimenter was blind to outcome assessment due to randomisation.

### Assessment of transplant success

Mice were excluded if there was technical failure (e.g. islets leaked from the portal vein into the abdomen) and if graft removal did not cause diabetes.

Transplants were considered successful if the average random fed blood glucose in the final week was below an arbitrary threshold of 15 mmol/l (~270 mg/dl). We have used this concentration in our previous work, as it allows clear differentiation from the diabetic blood glucose of ≥20 mmol/l [12]. The rate of endogenous beta cell regeneration and, therefore, exclusion was <10%.

### Immunohistochemistry

Grafts were collected and fixed in 10% formalin. Specimens were embedded in paraffin and cut into 5 μm sections. Antibodies for immunohistochemistry were rabbit anti-insulin (Cell Signaling Technology, Danvers, MA, USA) and anti-rabbit secondary antibody (Dako, Carpinteria, CA, USA) 1:100 in diluent. Sections were stained using a Dako autostainer, detected with a peroxidase substrate containing 3,3-diaminobenzidine (brown) (Dako) and counterstained with haematoxylin. For immunofluorescence, primary antibodies were rabbit anti-cleaved-caspase-3 antibody (R&D Systems, Sydney, NSW, Australia) and guinea pig polyclonal anti-insulin antibody (Dako) 1:100 in diluent. Secondary antibodies included anti-rabbit Cy3, anti-guinea pig Cy2 (Invitrogen, Thermo Fisher Scientific, Waltham, MA, USA) 1:100 in diluent and DAPI (Dako) 1:1000 in diluent. Images were taken with a Leica DM 5500 microscope and Zeiss AxioVision software (Oberkochen, Germany).

Calculation of beta cell area was performed using Image J (NIH freeware, www.nih.gov). Because the size of a graft varies along its length, every sixth section was quantified from the start of the graft to exhaustion of beta cells, and the trapezoidal method was used to calculate beta cell volume. Apoptosis was assessed by measuring cleaved caspase-3-positive, insulin-positive cell area in four sections spaced equally over the graft. We have previously reported on the antibodies used in this paper. Positive and negative controls were used and absence of signal with omission of the primary antibody was confirmed.

### Statistical analysis

For all figures, error bars indicate ± SEM. *p* values were calculated using either Microsoft Excel (Macquarie Park, NSW, Australia) or SPSS (v. 21.0; St Leonards, NSW, Australia).

Fisher’s exact test was used for categorical variables. When multiple comparisons were made, post hoc comparisons used ANOVA with Bonferroni correction. For two-group comparisons, unpaired two-tailed *t* tests with unequal variance were used. In all cases, a two-tailed *p* value of <0.05 was considered significant. Donors were excluded when all transplant sites failed.

## Results

### The highest success rate for mouse islet transplantation was obtained in the kidney subcapsular space

**Fig. 1:**
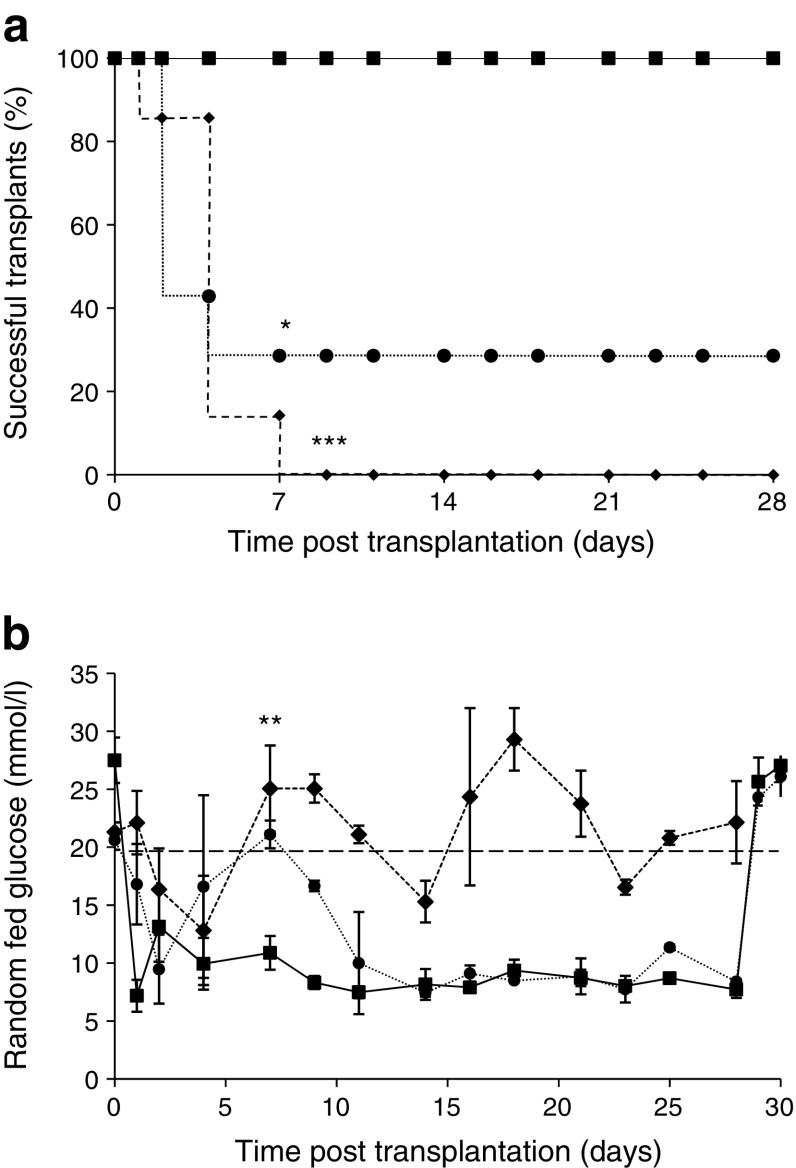
Diabetes cure rates and random fed blood glucose levels in mice implanted with murine islets into the kidney, liver or spleen sites. (**a**) Diabetes-free Kaplan–Meier graft survival curves. Recipients of 1:1 mouse islet transplants into kidney were cured in 100% of cases (solid line, squares, *n* = 5). Transplantation into liver capsule cured no mice (dashed line, diamonds, *n* = 6) and transplantation into splenic capsule (dotted line, circles) cured two of seven mice (*p < 0.05 and ***p < 0.001 vs kidney recipients). (**b**) Random fed blood glucose returned to normal by day 15 in two mice receiving islet transplants into the splenic capsule (dotted line, circles) but mice receiving transplants into the liver capsule failed to improve (dashed line, diamonds). **p < 0.01 vs control (transplant into kidney; solid line, squares)

C57BL/6 mouse islets were transplanted into diabetic C57BL/6 recipients in a 1:1 donor–recipient ratio. All control recipients (islets transplanted into the kidney subcapsular space [kidney]) were cured (*n* = 5). Two of seven mice (29%) receiving islets transplanted into the spleen subcapsular space (spleen) were cured and none of the six mice receiving transplants into the liver subcapsular space (liver) were cured (Fig. 1a). For transplants into both spleen and liver, recipients had either hypoglycaemic readings from day 2–4 or repeated high blood glucose resulting in the need for euthanasia within 1–2 weeks post transplant. Short-term hypoglycaemia is generally thought to indicate large-scale beta cell death. Success rates for spleen and liver sites (*p* = 0.028 and *p* < 0.001 by Fisher exact tests, respectively) were significantly lower than for kidney.

In the two successful transplants into spleen, random fed blood glucose levels were normal by day 15 (Fig. 1b). Blood glucose in those mice did not differ from those receiving transplants into kidney (9.2 ± 0.8 mmol/l vs 8.1 ± 0.3, *p* = 0.26). Islets transplanted into liver completely failed to normalise blood glucose in recipients (Fig. 1b, *p* = 0.009 vs kidney site). Overall, these results show that liver and spleen capsules are poor sites for transplantation of mouse islets.

**Fig. 2:**
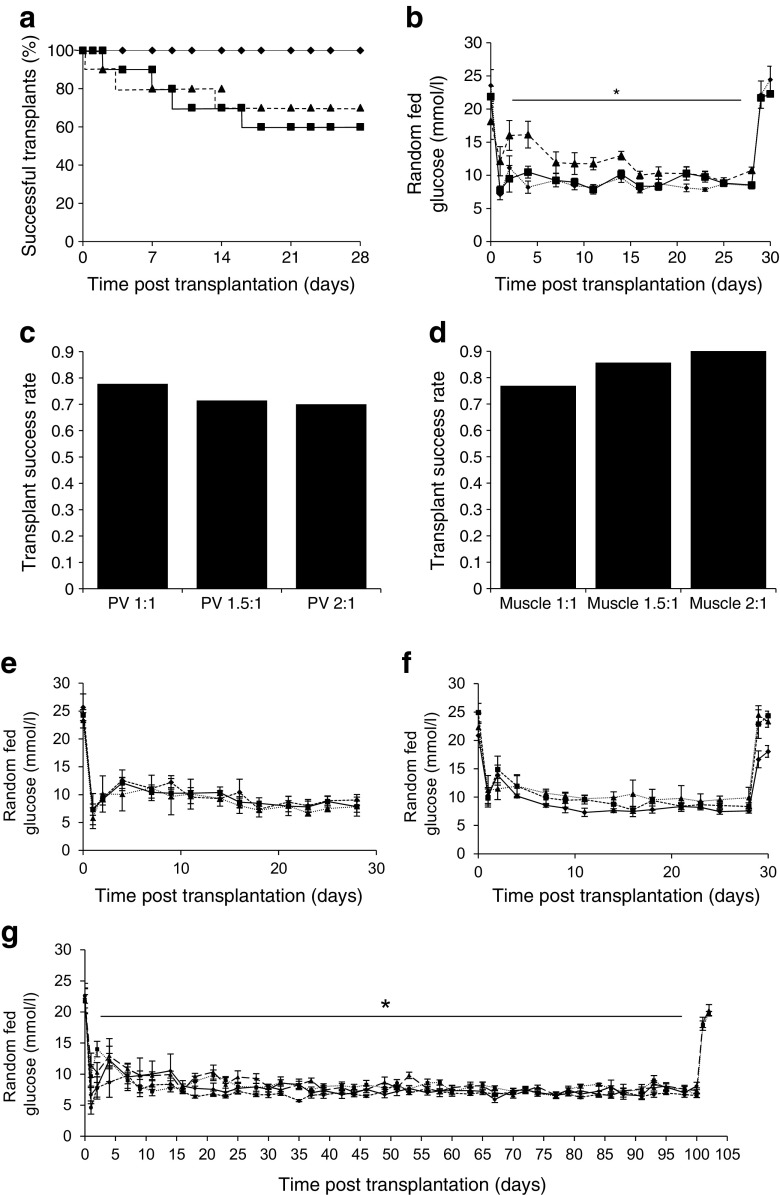
Diabetes cure rates and random fed blood glucose in mice implanted with murine islets into the kidney, muscle or portal vein sites. (**a**) Diabetes-free Kaplan–Meier graft survival curves. In control mice receiving islet transplants into the kidney (1:1), recipients were cured in 100% of cases (solid line, diamonds, *n* = 9). After transplantation of islets into muscle (dashed line, triangles, *n* = 10) and portal vein (solid line, squares, *n* = 10), recipients were cured in 70% and 60% of cases, respectively. (**b**) Random fed blood glucose in mice receiving islet transplants into kidney (dotted line, diamonds), muscle (dashed line, triangles) and portal vein (solid line, squares), *p < 0.05 for average of overall significant timepoints. (**c**) Success rates with increasing islet doses for transplantation into portal vein (7 out of 9 successful transplants for portal vein 1:1, 5 of 7 for portal vein 1.5:1 and 7 of 10 for portal vein 2:1). (**d**) Success rates with increasing islet doses for transplantation into muscle (10 of 13 successful transplants for muscle 1:1, 6 of 7 for muscle 1.5:1 and for muscle 2:1). (**e**) Random fed blood glucose in mice receiving islet transplants into portal vein, according to islet dose (solid line, black squares, portal vein 2:1; dashed line, black diamonds, portal vein 1.5:1; dotted line, black triangles, portal vein 1:1). (**f**) Random fed blood glucose in mice receiving islet transplants in muscle, according to islet dose (solid line, black diamonds, muscle 2:1; dashed line, black squares, muscle 1.5:1; dotted line, black triangles, muscle 1:1). (**g**) Long-term random fed blood glucose in mice receiving islets into portal vein and muscle sites, with two different donor–recipient ratios (solid line, black diamonds, portal vein 2:1; dashed line, black circles, portal vein 1:1; dotted line, black squares, muscle 2:1; long dashed line, black triangles, muscle 1:1) *p < 0.05 for average of overall significant timepoints

In a separate set of transplants, C57BL/6 mouse islets were transplanted in a 1:1 ratio into the right quadriceps muscle (*n* = 10) or portal vein (*n* = 10). A new set of contemporaneous controls, in which mice received transplants into kidney (*n* = 9), were used for comparison. Again, 100% of mice receiving transplants into kidney were cured (Fig. 2a). Mouse islets performed well in the muscle site: 70% of recipients were cured. Of the recipients transplanted at the portal vein site, 60% were cured. The random fed blood glucose in the final week of the 28 day study was 8.3 ± 0.2 mmol/l for mice transplanted at the kidney site vs 11.1 ± 0.7 mmol/l for muscle (Fig. 2b, *p* = 0.037, repeated measures ANOVA); blood glucose was 9.0 ± 0.3 mmol/l for portal vein (*p* = 0.15 vs kidney).

To determine the optimal dose of islets for each site to cure diabetes, we performed a further set of transplants into portal vein and muscle, with an increased graft size. C57BL/6 mouse islets were transplanted using donor-to-recipient ratios of 1:1, 1.5:1 and 2:1. Transplantation into the portal vein provided a good outcome, with a cure rate of 70–78%, although there was no clear improvement with increasing islet dose (Fig. 2c). In contrast, increasing islet dose for the muscle site further improved outcomes, with all 2:1 recipients being cured (Fig. 2d).

As shown in Fig. 2e, f, in the cured recipients, blood glucose was well-controlled. The 1:1 and 2:1 recipients were followed long term, and cure of diabetes was maintained (Fig. 2g).

### In mice, portal vein and muscle were the best clinical islet transplant sites for human islets

Human islets were transplanted using an adequate-mass model of 2000 IEQ into immunodeficient mouse recipients in one of five transplant sites: kidney, spleen, liver, portal vein or muscle. The control site was kidney. There is large inter-donor variability with human islets, so for every donor, at least one transplant into kidney was performed. If none of the control grafts (i.e. into kidney) taken from a human donor were successful, all data from that donor were excluded. With this caveat, recipients of transplant into kidney were cured in 78% of cases (14 of 18 transplants).

**Fig. 3:**
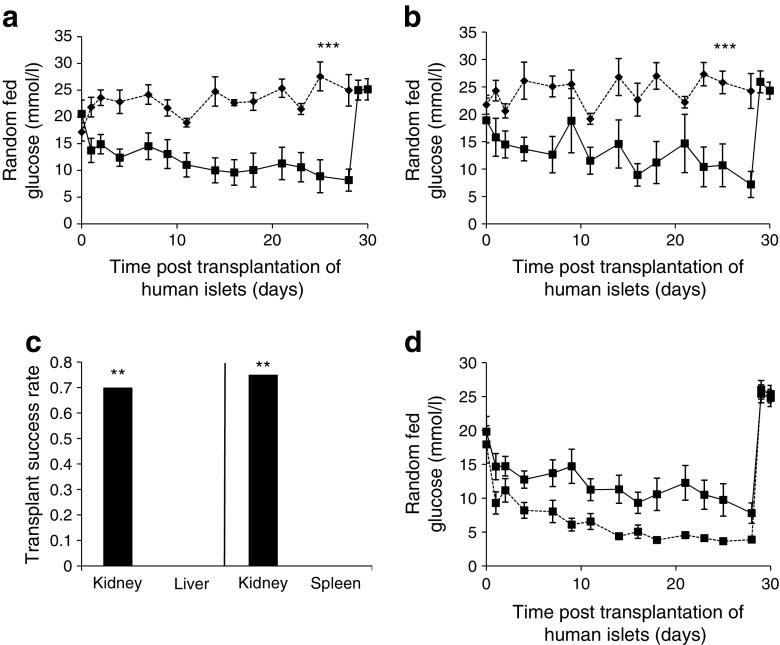
Comparison of diabetes cure and blood glucose levels with human islets transplanted into mouse kidney, liver or spleen sites. (**a**) Adequate-mass (2000 IEQ) transplants improved glucose levels in mice receiving transplants into kidney (solid line, squares) but not liver capsule (dashed line, diamonds) (*n* = 8, ***p < 0.001 kidney vs liver on day 28 post transplant). (**b**) None of the splenic capsule 2000 IEQ (dashed line, black diamonds) grafts (*n* = 6) achieved glucose control by day 28 (***p < 0.001 splenic vs kidney grafts on day 28 post transplant, solid line, black squares). (**c**) Mice receiving human islets (2000 IEQ) into kidney (control) were cured (glucose <15 mmol/l in the last week) in 70–75% of cases. Transplantation of islets (2000 IEQ) into liver capsule (*n* = 8) and splenic capsule (*n* = 6) cured 0% of mice at day 28 (**p < 0.01 liver vs kidney; spleen vs kidney, Fisher’s exact test). (**d**) Random fed blood glucose in recipients of successful islet transplants (2000 IEQ) into kidney capsule returned to normoglycaemic level by day 28 (solid line, squares, kidney all; dotted line, squares, kidney successful)

Figure 3a shows random fed blood glucose for recipients of islets from the same human islet donors transplanted into kidney vs liver capsule. All liver grafts failed (*n* = 8, *p* < 0.01 vs kidney, Fisher’s exact test). In the final week, the random fed blood glucose for mice receiving islet transplants into the liver vs kidney was 24.3 ± 1.4 mmol/l vs 9.4 ± 2.1 mmol/l, respectively (*p* < 0.001). Figure 3b compares blood glucose in mice receiving islet transplants into kidney vs spleen capsule, again with their matching control donors. Spleen-site transplants had all failed by 28 days (*n* = 6, *p* < 0.01 for success rate vs kidney, Fisher’s exact test). The blood glucose for spleen-site transplant recipients was 25.8 ± 1.4 mmol/l in the final week (*p* < 0.001 vs kidney). The transplant success rates of the liver and spleen transplant sites vs their donor-matched kidney-site controls are shown in Fig. 3c. Figure 3d shows the blood glucose curves for kidney grafts for all recipients (solid line) and for successful recipients only (dotted line).

**Fig. 4:**
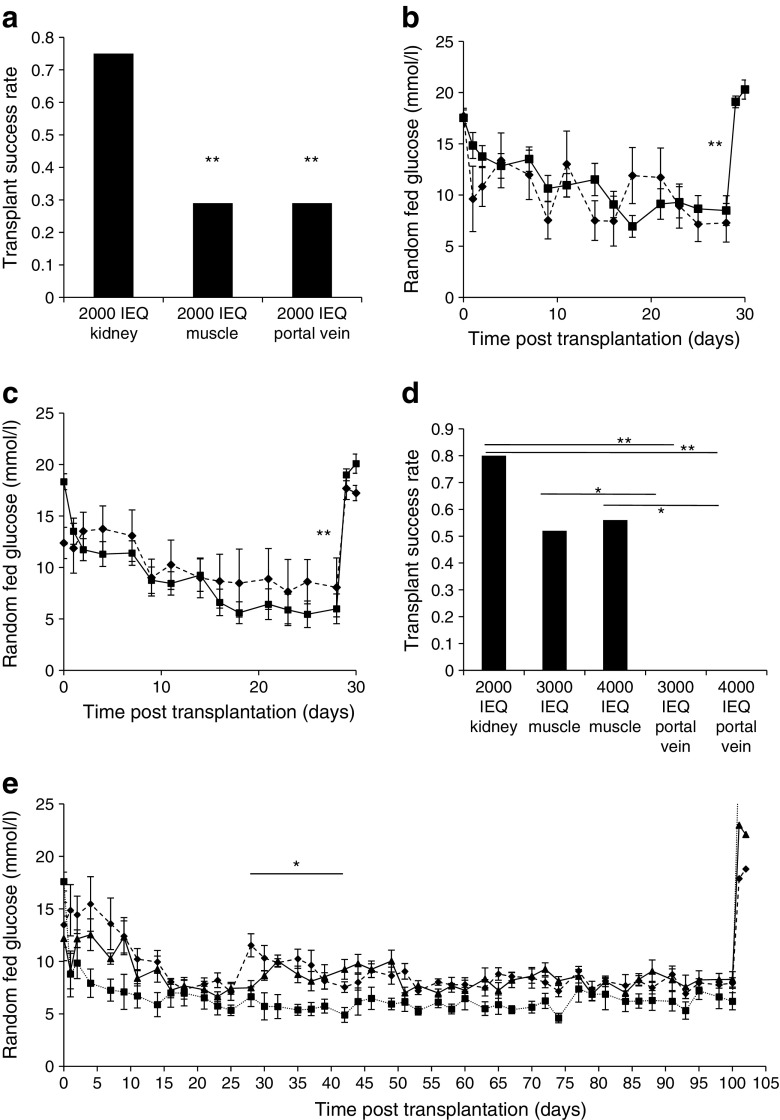
Comparison of diabetes cure and blood glucose levels with human islets transplanted into the kidney, muscle or portal vein sites. (**a**) The cure rate for mice transplanted with human islets (2000 IEQ) into kidney capsule (controls) was 75% (*n* = 24), similar to that achieved in previous experiments. At 2000 IEQ graft size, the cure rate achieved using either muscle (*n* = 14) or portal vein (*n* = 14) as the transplant site was 29% (**p < 0.01 vs kidney). (**b**) Cured recipients of islet transplants (2000 IEQ) into kidney (solid line, black squares) achieved better glucose control when compared with portal vein (dashed line, black diamonds) in the last week (**p < 0.01 for the last week, days 21–28 post transplant). (**c**) Random fed blood glucose in cured recipients of islet transplants (2000 IEQ) into kidney (solid line, black squares) and muscle (dashed line, black diamonds) (**p < 0.01 for the last week, days 21–28 post transplant). (**d**) In a new group of recipients of islet transplants (2000 IEQ) into kidney (controls), 8 of 10 (80%) were cured. Transplantation of higher doses of islets (3000 IEQ and 4000 IEQ) into portal vein cured 0 of 7 (0%) recipients (both doses, **p < 0.01 portal vein vs kidney). *p < 0.05 for indicated comparisons. Transplantation of higher islet doses into muscle cured 52–56% of recipients (8 of 16 for 3000 IEQ; 12 of 21 for 4000 IEQ). (**e**) Long-term random fed blood glucose levels were equivalent in cured recipients of 3000 IEQ (dashed line, black diamonds) and 4000 IEQ (solid line, black triangles) into muscle and recipients of 2000 IEQ into kidney (dotted line, black squares), except between days 28 and 42, when recipients with transplants at the kidney site had lower blood glucose, *p < 0.05 for days 28–42 post transplant

In separate experiments using seven new human donors, we compared muscle and portal vein transplant sites with the kidney. As shown in Fig. 4a, 75% of kidney grafts were successful. Muscle and portal vein grafts were each successful in four of 14 recipients (29% for both). As observed with mouse islets, these outcomes were inferior to kidney (*p* = 0.008). Among the cured mice, the average final-week random fed blood glucose in mice with portal vein grafts was 7.0 ± 1.6 mmol/l vs 4.9 ± 0.2 mmol/l in mice with kidney grafts (Fig. 4b, *p* < 0.01). For the muscle-grafted mice, the average final-week random fed blood glucose was 7.5 ± 0.7 mmol/l (Fig. 4c, *p* < 0.01 vs kidney graft). Together, these results indicate at least equivalent outcomes for muscle and portal vein transplant sites for human islets in murine recipients.

As with mouse islets, we then carried out a dose–response experiment to determine whether outcomes would improve with increased donor islet mass. Transplants were performed in muscle and portal vein at 3000 IEQ or 4000 IEQ with new human donors. The kidney grafts (2000 IEQ) produced similar results to other experiments, with eight of 10 mice (80%) being cured. Among the recipients of transplants at the portal vein site, many mice experienced ‘dumping’ with hypoglycaemia soon after transplant, usually considered to reflect high rates of beta cell death (Fig. 4d). None of the 3000 or 4000 IEQ transplants were successful at the portal vein site. In contrast, increasing islet dose improved muscle-site success rates from 29% to 52–56% (Fig. 4d). blood glucose in the cured recipients of islets transplanted into muscle were higher than in the cured recipients of transplants into kidney during days 28–42 but did not differ by the end of the study (Fig. 4e).

### Analysis of beta cell volume and apoptosis of grafted human islets in five sites, at 28 days after transplantation

**Fig. 5:**
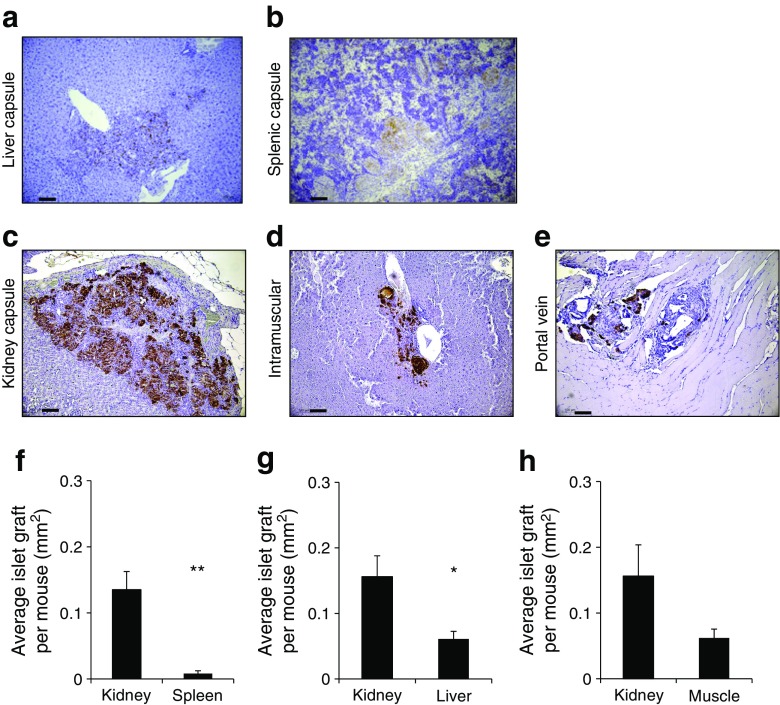
Beta cell volume in mice 28 days post transplantation of the human islet grafts. (**a**–**e**) Low-power images of the five transplant sites at 28 days post transplantation, showing insulin staining in brown. Grafts in the kidney capsule, muscle and portal vein showed normal insulin staining. Grafts sited in the liver and spleen capsule had less intense staining. Scale bars, 100 μm. (**f**–**h**) Quantification of beta cell volume from the images shown in **a**–**e**. At 28 days post transplantation, beta cell volume was substantially decreased in spleen and liver capsule transplant sites compared with kidney (*n* = 4 recipients per group; *p < 0.05 and **p < 0.01 vs kidney). The transplants in muscle had approximately 40% of the mass of the kidney capsule control, *p* = 0.14. Transplants into the portal vein could not be assessed due to dispersal throughout the liver

Beta cell volume was measured in the grafts at 28 days in the successful human islet transplants (2000 IEQ) into mouse kidney, liver, spleen and muscle (*n* = 4 mice/group). Figure 5a–e shows examples of insulin staining for each site. Transplants situated in the liver and spleen grafts showed less-intense insulin staining than those at the other sites. Graft beta cell volume is quantified in Fig. 5f–h. Consistent with the transplant failure, spleen had only 6% of the total graft beta cell volume compared with kidney. For recipients of islet transplants into the liver, beta cell volume was 36% that of kidney recipients. Transplants into the portal vein are diffused through the liver making it impossible to quantify the beta cell volume but there were clear areas of insulin-positive cells in the portal tracts. Muscle had 40% of the graft volume of their matched kidney controls.

**Fig. 6:**
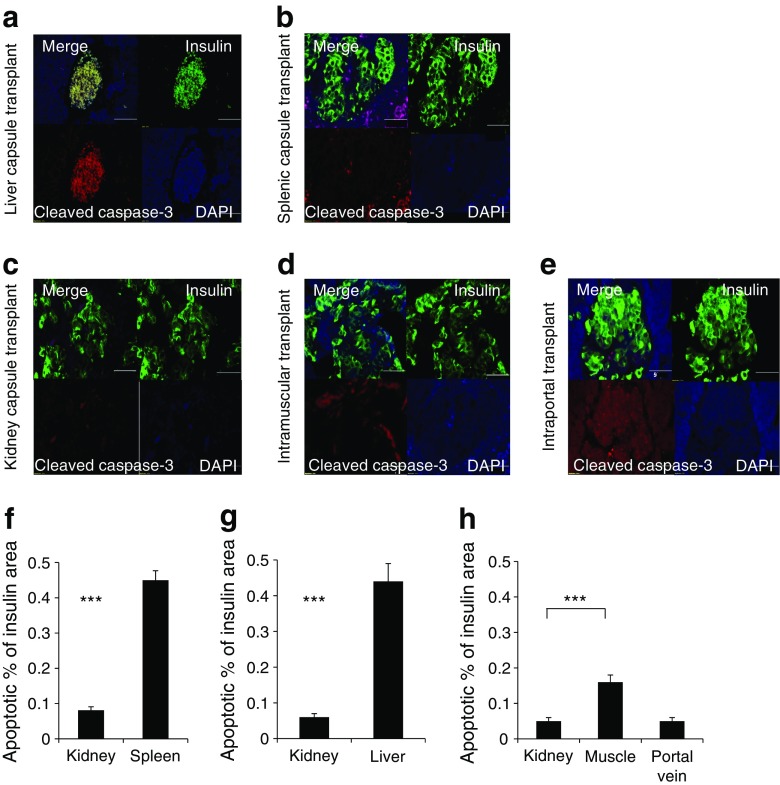
Apoptosis 28 days post transplantation in the human islet grafts in the five sites. (**a**–**e**) Representative low-power images of cleaved caspase-3 and insulin staining in each site 28 days post transplantation. Scale bars, 50 μm. (**f**–**h**) Quantification of apoptosis, as % of insulin-stained area in histology images shown in **a**–**e**. Quantification of cleaved caspase 3-positive and insulin-positive graft area was assessed at 28 days post transplantation. Grafts situated in the spleen and liver capsules showed a higher proportion of apoptotic area compared with kidney control (***p < 0.001). Although the apoptotic area in the muscle graft was greater than in the kidney graft (***p < 0.001), the apoptotic area was smaller in the portal vein and muscle grafts than in the spleen and liver capsule grafts

Apoptotic areas were assessed at day 28 following transplantation and expressed as a percentage of insulin-stained area when nephrectomy was performed to prove lack of endogenous beta cell regeneration. While this procedure would miss the initial spike in apoptosis, ongoing apoptosis at 1 month may be important for long-term transplant success. Representative low-power images are shown in Fig. 6a–e. Higher proportional apoptotic areas were seen in grafts situated in the spleen (47%) and liver (49%) when compared with kidney grafts (6–7%). Muscle and portal vein sites displayed lower apoptotic areas (16% and 5%, respectively) than spleen and liver (Fig. 6f–h). We note that spleen normally has many lymphocytes undergoing apoptosis; this is not relevant to islet transplant outcomes.

## Discussion

Pancreatic islet transplantation is a potential cure for type 1 diabetes but there are several limitations to the current practice of portal vein infusion. Here, we investigated the ability of human and mouse pancreatic islet transplantation into a variety of sites to cure diabetes in murine recipients. This study is unique in that comparisons of these different graft sites for human islets have not been performed previously.

Our first major finding was that implanting human and murine islets into the kidney subcapsular space yielded the best outcomes, with success rates of >75% and 100%, respectively. This was consistent with findings of our previous study [12]. The kidney has many advantages, including easy graft retrieval for exclusion of endogenous beta cell regeneration and histological assessment. It is worth noting that although the oxygen tension in islets placed beneath the kidney capsule is markedly lower than in native islets [14], this is true of all transplant sites investigated to date. Unfortunately, in humans, the kidney capsule has a relatively poor blood supply and is not readily amenable to creation of the >5 ml of anatomical space required to insert the islet mass [14, 36]. There are also theoretical concerns regarding damage to the kidney in individuals susceptible to diabetic nephropathy. However, attempts using the renal subcapsular space with modern islet transplant protocols have not been reported.

There is a published preclinical model in which a combined kidney and islet graft was transplanted [37]. The islet grafts showed resilience to hypoxic injury, the dual procedure saved time and there was a short time to islet graft function.

In the present study, we also tested the spleen and liver capsule as transplant sites, due to their good vascular supply and theoretical potential for direct laparoscopic graft visualisation. However, the liver and spleen-capsule transplants showed inferior results for both mouse and human islets.

Next, we found that the skeletal muscle and portal vein sites were equally successful in the 1:1 transplant model. The portal vein is currently the preferred site for clinical islet transplantation, although some investigators are now studying other sites [38]. The portal vein is relatively accessible but requires cannulation radiologically or under direct visualisation. This site delivers insulin to at least some of the portal system. The islets are exposed to intermittently high levels of immunosuppressive drugs, which are absorbed from the gut [17]. There is a risk of bleeding, portal hypertension and portal vein thrombosis [10]. Routine biopsies to monitor the graft are not feasible because the islets are dispersed throughout the liver. Furthermore, the liver can be a pro-thrombotic and pro-inflammatory environment [15, 16, 39].

Our success rates (29%) for human islets were not dissimilar to those reported for human clinical programmes. We note that most human recipients receive more than one islet transplant in the clinical setting. With transplants involving more than one donor, outcomes vary from 15% to 38% insulin independence at 1 year and 27% at 5 years after transplant [40, 41]. There is little data regarding single-donor transplant success rates for insulin independence. To address this question, we also performed dose–response studies to determine success rates with transplantation of increasing amounts of islets.

For the skeletal muscle site, we found that the success rate could be further increased by implanting more islets. With increasing islet dose, we achieved a 100% cure rate in recipients of mouse islet transplants into muscle (two donors, one recipient); the rate was 56% in recipients of human islets (3000 IEQ). In contrast, there was no improvement in outcome, with increasing islet dose, for recipients of transplants into the portal vein. Our data for intraportal transplantation show a similar success rate to that achieved in muscle grafts in 1:1 models, with no improved success observed with increasing doses as seen with muscle grafts.

For the kidney capsule, muscle and portal vein, there were no transplant failures up to 100 days in mice, which were normoglycaemic at day 28, showing that good graft function is maintained over the long term. Notably, glucose levels showed a continuing mild improvement up to day 60 for both muscle- and portal vein-sited mouse and human islet transplants.

One weakness of our study is that we could not remove the transplant to confirm diabetes recurrence in the portal vein group. However, this may have led to overestimation of the success rate of our portal vein group and does not undermine the conclusions.

The body weight of recipient mice may be a confounding variable in islet transplant studies. Loganathan et al [42] reported higher survival rates in recipient mice weighing ≥25 g. The weight range of our recipient mice was 20–25 g and generally we also found somewhat better results in heavier mice. However, the weight of recipient mice did not differ between the human islet transplant sites: 2000 IEQ into kidney capsule–23 g; 2000 IEQ into portal vein–23 g; 2000 IEQ into muscle–22 g, 3000 IEQ into portal vein–20.3 g, 4000 IEQ into portal vein–19.2 g, 3000 IEQ into muscle–21.6 g and 4000 IEQ into muscle–20.4 g. Our data also supported those of Loganathan et al [42], in that increased IEQ achieved better glucose control in muscle but not the portal vein. Because there is not a capsule like in the kidney, both portal vein and muscle sites lead to greater dispersal of islets. This dispersal may be beneficial to the recipient, although it makes quantification of beta cell volume more difficult. While it is not possible to quantify beta cell volume in the portal vein, individual islets and insulin intensity appeared to be similar to those in muscle.

The muscle as an islet transplant site has potential advantages, including access for biopsies [43]. In our study, we also noted shorter procedural time. There was a superior success rate compared with portal vein with increased graft mass. In cured recipients, long-term glucose control was similar for muscle and portal vein sites [19]. Our findings for muscle are supported by an earlier study showing successful transplantation of a larger volume of rat islets into muscle [30].

Other potential sites have also been investigated. Guan and colleagues showed good results for rat islets transplanted into the portal vein or omental pouch in the 1990s [44] (omentum is another potential site for islet transplants). Our study chose similar sites to Kim et al (2010) [20], however with emphasis on the importance of clinical adaptation. However, Kim et al reported that transplantation into the omental pouch produced a higher mortality rate and required a longer operative time, so we did not assess it in the present study. Epididymal fat pad and intestinal submucosa in large animals have been investigated for encapsulated islets with good success rates [45, 46].

Potential clinical relevance was achieved by using human islets and gaining an analysis of islet function by using immunodeficient recipient mice. This allowed us to examine engraftment at different sites, without the complication of immune rejection. While these mice have immune limitations, in our study transplant into muscle provided better glycaemic control than that found by Kim et al using C57BL/6 recipients. The use of an increased islet dose in the portal vein showed relatively poor outcomes, probably due to physical factors (i.e. increased mass of islets per blood vessel volume within the liver). Engraftment at the various tissue sites will be affected by blood flow, oxygen tension and tissue characteristics. However, it is important to note that engraftment may differ in humans and, further, may be influenced by immunosuppressive drugs.

Islet transplantation is a rapidly progressing field, with improvements in the isolation process, donor and recipient selection and very active investigation of optimal immunosuppressive protocols [3, 47, 48]. The optimal site for the transplant could have a major effect on long-term outcome as well as short-term complications, as has been seen in whole-pancreas transplantation [49–51]. Islet encapsulation technology is progressing rapidly and there are now implantable devices that can be inserted and allowed to revascularise prior to transplantation. It would be feasible to insert these devices under muscle capsules when the devices are optimised.

The intramuscular site avoids some of these issues and shows potential for successful outcomes for up to at least 100 days. The combination of good outcomes in human intramuscular autotransplantation case reports and these study results suggest that consideration of a multicentre human clinical trial is warranted.

## Abbreviations

IEQ: Islet equivalents
RAG-1: Recombination-activating gene 1
SCID: Severe combined immunodeficiency
